# Quality priorities related to the management of type 2 diabetes in primary care: results from the COMPAS + quality improvement collaborative

**DOI:** 10.1186/s12875-024-02641-9

**Published:** 2024-11-16

**Authors:** Dina Gaid, Guylaine Giasson, Isabelle Gaboury, Lise Houle, Géraldine Layani, Matthew Menear, Véronique Noël de Tilly, Marie-Pascale Pomey, Brigitte Vachon

**Affiliations:** 1 School of Rehabilitation, Faculty of Medicine, Université de Montréal, CP 6128 Succursale Centre-Ville, Montreal, QC H3C 3J7 Canada; 2 Department of Family Emergency Medicine, Faculty of Medicine and Health Sciences, Centre de recherche Charles-LeMoyne (CR-CRCLM), Université de Sherbrooke – Campus de Longueuil, Longueuil, QC Canada; 3 Department of Family and Emergency Medicine, Faculty of Medicine and Health Sciences, Centre de recherche Charles-LeMoyne (CR-CRCLM), Université de Sherbrooke Campus de Longueuil, Longueuil, QC Canada; 4 Institut national d’excellence en santé et en services sociaux (INESSS), Montreal, QC Canada; 5 Department of Family Medicine and Emergency Medicine, Université de Montréal, Montreal, QC Canada; 6 Centre de recherche du Centre hospitalier universitaire de l’Université de Montréal, Montreal, QC Canada; 7 Department of Family Medicine and Emergency Medicine, Université Laval, Quebec, QC Canada; 8 VITAM – Centre de recherche en santé durable, Quebec, QC Canada; 9 Convergence Santé, Montréal, QC Canada; 10 Public Health School, Department of Management, evaluation and health policy, Faculty of Medicine, Université de Montréal, Montreal, QC Canada; 11 Centre de recherche du CIUSSS de l’Est de l’Île de Montréal, Montreal, QC Canada

**Keywords:** Type 2 diabetes Mellitus, Interprofessional collaboration, Primary care, Quality improvement collaborative, Quality of care

## Abstract

**Background:**

This study aims to describe the main type 2 diabetes mellitus (T2DM) quality improvement (QI) challenges identified by primary care teams in the province of Quebec who participated in the COMPAS + QI collaborative.

**Methods:**

A qualitative descriptive design was used to analyse the results of 8 COMPAS + workshops conducted in 4 regions of the province between 2016 and 2020. Deductive content analysis was performed to classify the reported QI priorities under the Consolidated Framework for Implementation Research domains; and proposed change strategies under the Behavior Change Wheel (BCW) intervention functions.

**Results:**

A total of 177 participants attended the T2DM COMPAS + workshops. Three QI priorities were identified: (1) lack of coordination and integration of T2DM care and services; (2) lack of preventive services for pre-diabetes and T2DM; and (3) lack of integration of the patients-as-partners approach to support T2DM self-management. The proposed QI strategies to address those priorities were classified under the education, training, persuasion, habilitation and restructuring BCW intervention functions.

**Conclusion:**

This study provides insights on how QI collaboratives can support the identification of QI priorities and strategies to improve T2DM management in primary care.

## Introduction

Diabetes Mellitus (DM) is one of the most common chronic diseases worldwide, with a global prevalence (between 20 and 79 years old) estimated at 10.5% (536.6 million people) [1]. In Canada, 29% of the population currently live with DM or prediabetes, a percentage that is expected to rise to 33% by 2025 if current trends continue [2]. DM is also a very costly disease; global diabetes-related health costs were estimated at 966 billion USD in 2021 and were predicted to reach 1,054 billion USD by 2045 [1]. 

Type 2 Diabetes Mellitus (T2DM) is a chronic metabolic disorder characterized by persistently high blood glucose levels due to the body’s inability to effectively use insulin [3]. T2DM is often linked to lifestyle factors such as obesity, physical inactivity, and poor diet [4]. and is a major risk factor for coronary heart disease and a leading cause of disability and death globally [5]. Frequent and continuous T2DM follow-up is recommended to reduce diabetes-related complications, support self-management, and help patients achieve their care goals [6–8]. According to the American Diabetes Association guidelines [9] and the Canadian Diabetes Association guidelines [10], monitoring glycemic status should be at least two times a year in patients who have stable glycemic control, and should be at least quarterly in patients whose therapy has recently changed and/or who have unstable glycemic control. Treatments should be prescribed to control hemoglobin (HbA1c), blood pressure (BP), and cholesterol in respect of patient preferences and goals, and functional status [6, 9]. 

Despite well-defined best practices and the availability of both pharmacologic and nonpharmacologic treatments [11–13], substantial quality gaps exist in the management of T2DM [14–20]. The 2013 Diabetes Mellitus Status in Canada (DM-SCAN) study, which included 5,123 individuals with T2DM, reported low rates of achievement on several diabetes outcomes targets: only 50% of patients surveyed met the defined HbA1c target (A1C 7.0%); 36% met the BP target (130/80); 57% met the low-density lipoprotein-cholesterol (LDL-C) target (< 2.0 mmol/L); and only 13% met al.l 3 targets [21]. Most of the care for Canadians living with T2DM is provided in primary care [7, 22] but delivering evidence-based T2DM care in these settings remains a persistent challenge [23]. A systematic review by Rushforth et al. [24] reported several barriers for the adoption of diabetes guidelines in primary care, including large workloads and time pressures, the lack of information and protocols for diabetes management, a lack of continuity of care, and limited continuing education opportunities for clinicians. Other notable barriers included clinicians’ lack of confidence in their knowledge of T2DM guidelines and a lack of clarity around roles and responsibilities in diabetes management across primary and secondary care providers. [24] Another recent review [25] has identified challenges related to linkages with community resources, continuity of care, coordination in referral systems within the health care team, interactions between patients and clinicians, language barriers, and limited patients-healthcare providers interactions to be factors affecting the quality of diabetes care.

Despite these many challenges, quality improvement (QI) programs can support measurable improvement in the quality of diabetes care and patient outcomes [26, 27]. For example, 66 controlled studies showed that QI interventions can significantly lower HbA1c by a mean 0.41 − 0.62% (95% CI 0.29–0.54) [27]. The evidence is clear that QI interventions directed toward the entire system of chronic disease management were found to be associated with the largest improvement effects irrespective of baseline HbA1c [27]. Quality improvement collaboratives (QICs) are multi-faceted interventions that can promote clinical practice guidelines uptake by clinicians and support their engagement in QI [28–30]. QICs involve multidisciplinary groups of professionals and managers who participate in a structured process to identify practices gaps and exchange strategies to be implemented to improve the quality of services [31, 32]. Although models vary, QICs generally deal with a specific health care topic, involve multiple clinical teams from multiple sites, incorporate a series of structured activities (e.g. meetings, workshops, audit and feedback, activities to promote collaboration) and encourage rapid, small-scale change based on a model of improvement [31, 33]. A recent study by Wells [34] examined the evidence on QICs from 64 studies and found that significant improvement in service quality was observed in 83% of studies, including 85% of primary care studies.

To support the improvement of chronic disease management in primary care settings in the province of Quebec, Canada, the Ministry of Health and Social Services is presently implementing a large-scale QIC initiative called the COMPAS + program. The National Institute of Excellence in Health and Social Services (INESSS), the province’s provincial advisor on clinical excellence in health and social care, and the Ministry jointly coordinate COMPAS+. The core objectives of the program are to enhance interprofessional collaboration, improve service quality for priority chronic illnesses, and actively engage patients and primary care providers in quality improvement [35]. The key innovations of COMPAS + include its utilization of population-based data to offer performance feedback on chronic illness management, its ability to encourage critical reflection on care and services delivery, and its ability to assist in the development and implementation of quality improvement action plans through cooperative problem-solving [35, 36]. In order to support local QI projects, the COMPAS + program activities include participation in a one-day reflective workshop,, and providing facilitation to a local QI team during one to two years to support change implementation. Additionally, patients-as-partners actively participate in the various QIC activities. This gives them the chance to share their knowledge gained from their experiences and help identify, in conjunction with primary care teams and healthcare managers, the most significant obstacles to managing chronic diseases and the necessary solutions to enhance services and care experiences. In order to enhance T”DM primary care services, four regions in the province of Quebec participated in the COMPAS + initiative between 2016 and 2020. This study aims to describe the key QI challenges reported by the participating teams, as well as the strategies they suggested to enhance primary care services for diabetes in Quebec.

Thus, the project aimed to answer these four specific research questions:

1) What are the perceived gaps for improvement in the management of T2DM in primary care in Quebec?

2) What are the main root causes perceived to give rise to quality gaps in the primary care management of T2DM?

3) What change strategies are proposed to reduce these quality gaps?

## Materials and methods

### Design

To answer these research questions, we conducted a qualitative descriptive study to compare and combine the results from the 4 Integrated Health and Social Services Centres (CISSS) et 8 local services networks (LSN) that received the COMPAS + intervention for T2DM between 2016 and 2020.

### Data collection

A full-day COMPAS + workshop employs three main strategies to get people engaged with QI: (1) a feedback intervention to interactively introduce performance indicators by relating them to the recommendations outlined in diabetes clinical practice guidelines; (2) reflective exercises in small and large groups in order to establish a shared understanding of performance indicators and the most significant quality gaps; and (3) a collaborative problem-solving and action planning process involving small groups of eight to ten individuals who identify priority QI challenges and their root causes, propose workable solutions, and negotiate the content of their QI action plans. Experts in change management and quality improvement, together with INESSS, lead these sessions. In order to help local action plan execution, they also offer four facilitation follow-up meetings, which may take place up to 24 months after the workshop. You may find more information on the COMPAS + QIC in previously published articles [37–40]. Following every session, the facilitator (VNT) produced an extensive report. The arrangements and procedures of each workshop are detailed in these reports. Additionally, they include a description of the suggested action plans, the list of suggested change strategies, the findings of the root-cause analysis activities, the QI priorities identified, and the outcomes of the small and large group reflection exercises. The main findings of the follow-up visits and the systematic implementation of the action plans were also recorded in the notes made by the facilitator. Our team had no access to clinical data, such as HbA1C or blood pressure, since this information is not available in Quebec’s healthcare administrative data. Analysis was performed on eight workshop reports, eighteen action plans, and seven reports from follow-up visits.

### Data analysis

Deductive content analysis was performed. Priority problems were organized in categories and root causes were classified according to the Consolidated Framework for Implementation Research (CFIR) domains [41]. Solutions or strategies proposed by participants were organized and categorized into the intervention function types described in the Behavior Change Wheel [42]. Action plans, implemented locally after the workshops, were analyzed separately. All documents were uploaded and coded into the NVivo software (QSR International Pty Ltd. Version 12, 2018). Tables were also created in Excel sheets to compare data extracted from each region. Co-coding was performed by two members of the research team (GG, BV). Three members of the research team (BV, GG, IG), one workshop facilitator (VNT) and one patient mentor (LH) (who had attended all the diabetes workshops) met three times to validate the data analysis process and the results table produced.

## Results

### Characteristics of participants

A total of 177 persons participated to the T2DM COMPAS + workshops in the four regions. They included primary care professionals from multiple different disciplines, patients-as-partners and managers. Details are provided in Table 1.

**Table 1:** Description of workshop participants in each region

Region	A	B	C	D	Total
Number of workshops in the region	2	2	3	1	**8**
Number of workshop participants	40	44	63	30	**177**
Types of participants					
• Family physicians	7	7	8	6	**28**
• Nurses	18	18	15	10	**61**
• Nutritionists	2	5	7	2	**16**
• Pharmacists	4	3	6	2	**15**
• Social workers	0	1	4	2	**7**
• Kinesiologists	0	0	0	1	**1**
• Internists	0	1	1	1	**3**
• Patient partners	2	3	6	2	**13**
• Managers	5	6	15	4	**30**
• Administrative staff	1	0	0	0	**1**

### A. Key T2DM Quality Improvement gaps and Main Root causes

**Fig. 1:**
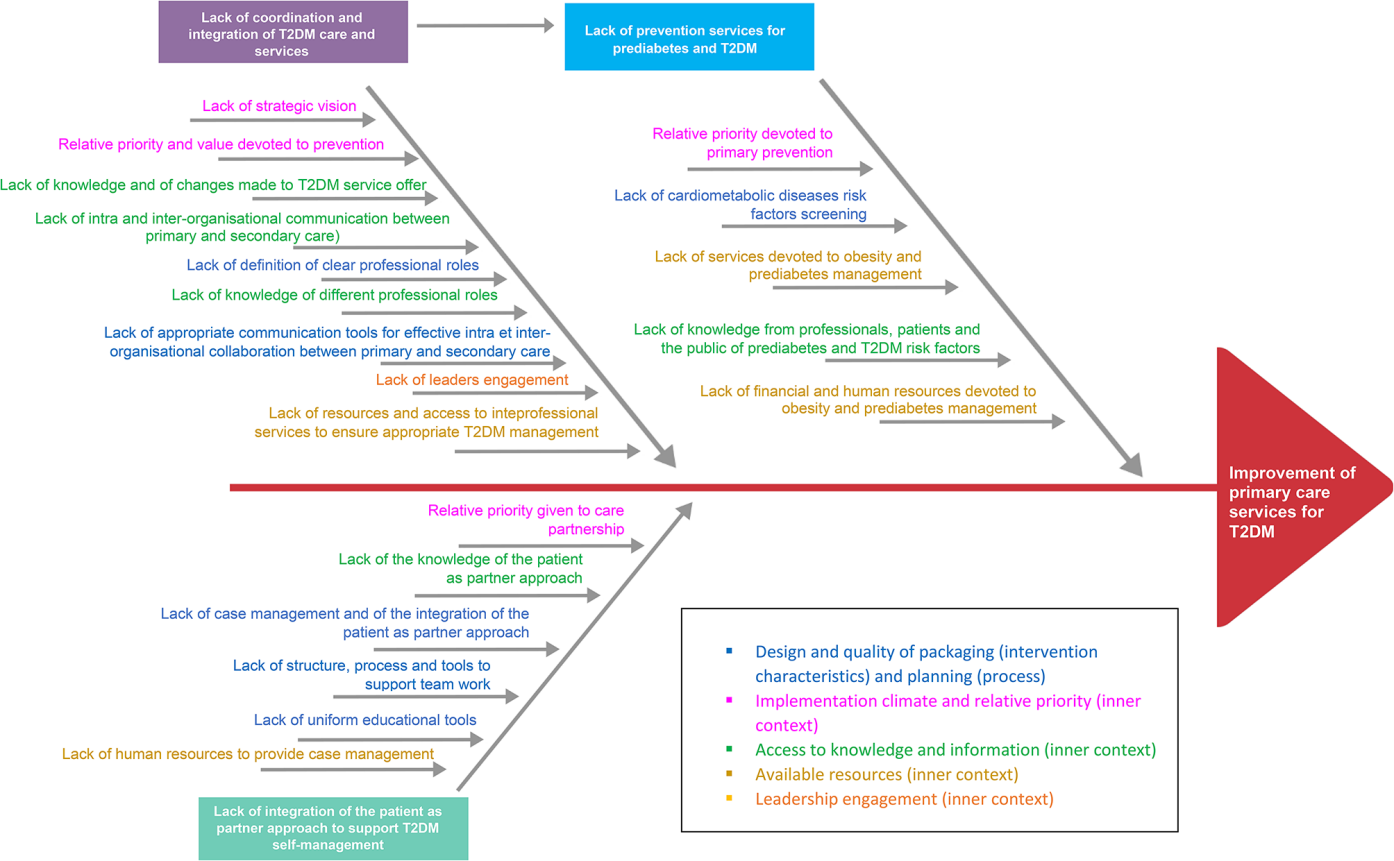
The quality gaps identified by COMPAS + workshop participants for improving the quality of T2DM care

Figure 1 describes the three quality gaps identified by COMPAS + workshop participants for improving the quality of T2DM care: (1) lack of coordination and integration of T2DM care and services; (2) lack of preventive services for pre-diabetes and T2DM; and (3) lack of integration of the patients-as-partners approach to support T2DM self-management. The analysis of the qualitative results is presented in the form of a fishbone diagram. This type of figure is used here to represent the main causes of why diabetes primary care services are currently sub-optimal, where the head of the fish represents the target for improvement and the branches represent the root causes. Root causes were classified into CFIR constructs. Only seven constructs of the framework were used to classify these factors: design quality and packaging (intervention characteristics), planning (process), implementation climate (inner context), relative priority (inner context), access to knowledge and information (inner context), available resources (inner context), leadership engagement (inner context). Different colors were used, in the diagram, to distinguish factors belonging to different CFIR constructs. As can be seen, similar root causes were found to influence the three main quality gaps.

#### A.1. Unfavorable implementation climate and relative priority to improve T2DM services

Implementation climate, referring to the capacity and receptivity for change, was found to be one of the main barriers influencing the achievement of all three quality gaps Root causes such as lack of strategic vision and relative priority and value devoted to primary and secondary prevention were described by participants as influencing the importance given to diabetes care and self-management. This implementation climate was also described as influencing human and financial resources devoted to the prevention of obesity and diabetes and the availability of appropriate services from different professionals, especially nutritionists and nurses, for preventive care, early detection of the disease, and early management of T2DM before complexity and complications.

#### A.2. Lack of T2DM knowledge to engage in the full scope of practice

Not surprisingly, lack of knowledge was also found to be an important barrier to the delivery of evidence-based T2DM primary care services. Participants reported a lack of knowledge and understanding of all team members’ professional roles. This negatively impacted interprofessional collaboration and timely referrals to appropriate services. Lack of familiarity with new T2DB evidence-based risk factors was associated with an important lack of screening of these factors and inertia towards the management of obesity since very few services are available to support at-risk patients. Furthermore, participants also reported a lack of knowledge from most professionals on the ways in which to integrate the patients-as-partners approach, the importance of self-management support and especially motivational interviewing techniques into routine primary care practices. This was considered as having important consequences on the identification and achievement of patients’ self-management goals which can greatly support them in improving glycemic control and preventing T2DM complications.

#### A.3. Insufficient T2DM proactive care and services planning

Participants also recognized the need to harmonize teamwork processes but observed a lack of clear leadership responsible for planning this improvement process. Communication tools were also viewed as insufficient and ineffective to ensure the provision of integrated care and help patients navigate the healthcare system and access services in a timely matter. A lack of service coordination was also found to influence the provision of T2DM prevention services since resources of the healthcare system were devoted to providing services to diagnosed cases. Participants also expressed problems related to a lack of structure, processes, and tools to coordinate teamwork effectively to provide case management services, centered on patients’ expressed needs and priorities. Lack of uniform, validated educational materials that could be used by all members of the primary care team, but also by professionals delivering self-management education and support to the same patients outside these teams and in specialty diabetes care, was named as important to facilitate the provision of education and support and increase patients’ engagement in self-management.

### B. proposed QI strategies and locally implemented strategies

During COMPAS + workshops, participants identified multiple QI strategies and solutions that could be implemented to improve T2DM primary care. These strategies were analyzed to identify which categories of the CFIR constructs they were expected to change and to which main behaviour change techniques they were associated (Table 2).

**Table 2:** QI strategies proposed by workshop participants

CFIR constructs	Proposed strategies classified into BCW intervention functions	Examples of implemented strategies
**Access knowledge and information**	**Education** - Educational activities and material to improve knowledge of new evidence-based risk factors - Standardization of training for professionals from all disciplines to improve coherence of patient education throughout the continuum of care made available on the provincial training online platform - Educational activities and material to improve use of the patient-partner approach and effective strategies to engage patients in self-management - Educational material to improve patients’ knowledge of available health and social services and community resources Persuasion - Use social influence and media to increase population awareness of diabetes risk factors and importance of health habits - Disseminate the importance of assessing new risk factors (ex: waist circumference) - Develop key and attention-grabbing messages - Use local champions to provide education **Training** - Provide training on therapeutic relationship establishment, identification of patients’ personal objectives and assessment of patients’ health beliefs and attitudes	Training delivered to all nurses working in family medicine groups Development of online and in-person educational programs adapted to patients’ preferences, availability and work schedule
**Design quality and packaging** **Planning** **Available resources**	**Habilitation** - Design and implement a clinical care pathway - Define and implement the case manager role to improve continuity of care - Design and improve communication tool - Implement tools developed by INESSS - Integration patient activation action plan (electronic version) - Regular updates of resources directory - Design a tool to screen risk factors for diabetes that would be integrate within clinical note template - Follow quality indicators **Restructuring** - Create a registry to improve patient follow-up - Make the nurse responsible of non-registered patients - Prioritize registration of patients living with diabetes	Development of a new referral form for physicians and other professionals to directly refer their patients to the diabetes education and support program Development of common tools used by hospital-based services, family medicine groups and the diabetes education and support program Creation of a service corridor for patients not registered with a family physician or for urgent consultations
**Implementation climate** **Leadership engagement**	**Habilitation** - Create a continuous quality improvement committee led by a champion and composed of professionals from all disciplines and patients-partners - Provide change facilitation and support - Create opportunities for professionals to network, discuss their roles and clinical cases	Creation of a consultative committee to develop a common and expanded care pathway including the participation of patient as partners Development of a protocol to clarify the role of the diabetes education and support program and transition mechanisms between primary and secondary care services

#### B.1. Education, training and persuasion

To improve access to evidence-based knowledge and information, educational activities, delivered simultaneously to professionals from all disciplines, were proposed to increase the standardization of training in the province of Quebec and increase the coherence of care delivery throughout the continuum of care. These continuing education interventions should be made available on an existing provincial online training platform. Even if most professionals are already aware of the importance of adopting patient-centered interventions, more training on the patients-as-partners approach and motivational interviewing techniques was deemed essential. Local champions should be identified to support and provide education and training sessions. For example, development of online and in-person educational programs adapted to patients’ preferences, availability and work schedule have been implemented in some settings. Furthermore, access to standard and high-quality educational materials for patients, available in the healthcare system and in the community, was considered essential to increase the dissemination of information on T2DM and its prevention. Finally, interventions using persuasion as their main action mechanism were recommended to increase population awareness of diabetes risk factors and the importance of health habits, such as developing key and attention-grabbing messages and social influence and media.

#### B.2. Habilitation and restructuring

To improve the design, planning and delivery of services, participants recommended strategies that were classified in the habilitation and restructuring behavior change technique categories. First, it was considered essential to improve the design and implementation of T2DM clinical care pathways to increase integration of services within primary care and with secondary care also. Tools developed by INESSS to promote greater case management and increase use of standard medical protocols by physicians in collaboration with nurses and pharmacists are required to be routinely used by all to improve interprofessional collaboration. The development of tools that can support self-management, such as a screening tool for risk factors, patient activation action plans, a resources directory easily available to increase referrals to community organizations, should be prioritized. One implemented example is the creation of a service corridor for patients not registered with a family physician or for urgent consultations. Participants also recommended strategies to increase engagement in QI, such as monitoring of quality indicators, development of a T2DM patient registry to ensure accurate follow-up and implementation of a system to facilitate priority registration of patients diagnosed with T2DM with a family physician or with a nurse practitioner.

Strategies to improve climate and leadership within the organizations were also proposed, such as setting up an organizational committee responsible for T2DM improvement, providing QI change management, facilitation and support and creating more opportunities, such as the one provided by the COMPAS + workshop, to network and discuss their professional roles.

### C. Example of a QI project implemented by a participating site to improve integration and coordination of T2DM services

During the COMPAS + workshop, participants reported a lack of collaboration between family medicine groups (FMGs) and services provided by the Chronic Disease Support and Intervention Center (CDSIC) of their region. This center offers short-term individual follow-up and group education sessions on diabetes self-management provided by an interprofessional team composed of nurses, nutritionists, kinesiologists and social workers. During the reflective workshop, a lack of awareness of services and of the ways in which patients could be referred to these services was identified as an obstacle to better integration of primary care services available. Following the workshop, the manager responsible for the CDSIC decided to lead a QI project in collaboration with patients-as-partners. The aim of the QI project was to increase awareness of the FMG nurses and physicians of services available, improve confidence in services provided, and adapt service offers based on patients’ and FMG professional’s perceived needs. An advisory committee was created to improve referral mechanisms (ensure that both patients and professionals can refer patients to the CDSIC); the definition of shared roles between FMG nurses and the CDSIC team (definition of who is responsible for what and for how long), the capacity of the CDSIC to see urgent cases in priority and improve the service offered to facilitate patient access outside regular working hours and via virtual group sessions.

## Discussion

The COMPAS + QIC has allowed our team to identify what are the main perceived quality gaps we need to overcome to improve the quality of T2DM primary care services in Quebec. The three most important quality gaps identified were: prediabetes and T2DM prevention, coordination and integration of care and services, and ensuring that patients were real partners in their care. Interestingly, these results are similar to other studies conducted in developed and underdeveloped countries [40] and important efforts should be made by healthcare systems to address these challenges since T2DM has become the modern preventable pandemic impacting more than 10% of the global population and costing hundreds of billions of dollars each year [43]. 

There was wide agreement among COMPAS + workshop participants that T2DM prevention efforts in the province of Quebec were insufficient. Increasing prevention efforts for T2DM would bring about important public health benefits, such as lowering rates of cardiovascular disease, renal failure, blindness, and premature mortality [10]. Even if it is well known that preventive measures are effective and cost-effective to prevent diabetes and its complications [9, 10], it has been challenging for primary care professionals to integrate preventive practices in routine care [43]. In a narrative systematic review of factors affecting diabetes prevention in primary care settings, Messina et al. [44] found multiple studies reporting that family physicians were often unaware of risks of pre-diabetes progressing to diabetes, considered prevention as expensive, time consuming, and as not a priority in consultations with patients when faced with other competing health priorities. However, this review also identified studies, from European countries and Australia, where diabetes prevention was said to be integrated into the role of primary care professionals and was part of routine checks and care since prevention was driven by policy mandate in these countries. [44] Another barrier to increasing prevention is the attitude of healthcare professionals who often downplay the seriousness of diabetes and think that people at risk of T2DM will not engage in lifestyle changes since they consider it too difficult [40, 45]. As mentioned by Haseldine et al. [45], achieving high participation in prevention programs is challenging since patients also often do not attend them. Lack of awareness and fear of diabetes was found to influence patients’ motivation to attend. This is also in line with our participants’ perspectives that the public generally lacks knowledge about diabetes and that this is an important barrier to T2DM prevention. Furthermore, increased immigration in the province of Quebec can also require more prevention efforts since certain ethnic groups, including African, Arab, Asian, Hispanic, Indigenous and South Asian peoples, are more at risk of metabolic syndrome and diabetes [10]. Providing more healthy behavior interventions, nutrition therapy, physical activity and pharmacotherapy to at-risk individuals is essential [10]. In the United States, a national diabetes prevention program (https://www.cdc.gov/diabetes/prevention/index.html) was implemented to provide a population-based intervention at low-cost across America [46]. It consists of four core elements: (1) a trained workforce of lifestyle coaches; (2) national quality standards supported by the CDC Diabetes Prevention Recognition Program; (3) a network of program delivery organizations sustained through coverage; and (4) participant referral and engagement. This population approach facilitates access for everyone to a lifestyle change program. These diabetes prevention programs can also be accessed more widely in the community since they are not only provided by healthcare professionals, but also by trained coaches with various educational and experiential backgrounds [47]. As mentioned in a review on public health approaches for T2DM prevention, large-scale and population-wide strategies are needed [48]. Improved public understanding is critical for the early diagnosis as well as prevention of diabetes. It would be relevant for provincial health authorities in Quebec and other parts of Canada to adopt and implement similar population-based approach to prevention. On the other hand, our participants proposed that mass media campaigns be used to increase public awareness of the risks of developing diabetes and complications associated with T2DM. Awareness campaigns have the potential to change misperceptions around T2DM as a non dangerous condition and educate people on the simple steps that can be taken to meaningfully prevent or manage this chronic condition [49]. Such mass media strategies and prevention programs should be co-designed by public and the partners working together [49, 50]. 

Coordination and integration of T2DM care and services were also identified as a main quality gap that needs to be overcome. The Diabetes Canada practice guidelines has a whole chapter describing the ways in which diabetes services should be organized based on best available evidence [10]. Our system struggles to make these recommendations mandatory, and many patients remain without access to optimal follow-up even if resources are available within FMG, in secondary care or in the community. Patients should be able to receive self-management education and support from an interprofessional team, especially from a nutritionist and nurse trained in diabetes [49]. However, as mentioned by our participants, confusion in the definition of professional roles and how members of the interdisciplinary team should work together remains [51, 52]. This lack of structure, knowledge and communication tools reduces access to optimal care. In a recent systematic review on QI strategies for diabetes care [27], case management, team structure changes, patient education, and the promotion of self-management appeared to be the most effective quality improvement strategies to implement in practice [27]. Combining these different strategies with reminders and electronic patients’ registries was found to lead to significant improvement in blood sugar control. To improve care coordination, integration and better definition of professional roles, clinical care pathways can be developed. Integrated care pathways are designed to guide practice regarding optimal approach to improve care by reducing variations in clinical practice and promoting efficient use of health care resources [53]. Nevertheless, clinical care pathways implementation requires both healthcare professionals and managers engagement [54]. This leadership was also considered, by participants of this study, as a barrier to services quality. A review of 32 studies of integrated care interventions for type 2 diabetes, published in 2016, found that most facilitators to the implementation process were staff involvement in decision-making and planning, the ability to recruit committed staff and ensure buy-in, good leadership, and intra- and inter-practice cooperation and sharing of resources [55]. Improving middle managers’ leadership in the healthcare system should be a priority because it is essential to increase professionals motivation to adopt and use clinical care pathways and best practice guidelines recommendations. In Quebec, one example of this is the limited use of standardized medical protocols developed by INESSS [56] at the provincial level to promotes evidence-based practice and efficient use of resources in the health and social services sector. However, improving middle managers’ leadership may require providing training leadership programs to increase their capacity to empower their team in the adoption of best interprofessional practices [57, 58]. 

One of the most striking gaps reported in this study is the lack of integration of the patients-as-partners approach. In the province of Quebec, initiatives at various levels are being undertaken to promote care that is delivered in full respect of patients’ preferences and values and that they are fully engaged in shared decision-making.[39] Even if continuing education opportunities for healthcare professionals provides teaching on the approach, it seems difficult for the system to adopt this approach accordingly. In other jurisdictions, traditional paternalistic models of care remain common and patient-provider interactions remain centered on information transfer with limited opportunities to discuss social and psychosocial issues related to the management of T2DM [59]. Motivational interviewing is recommended to support lifestyle changes and self-management of diabetes and many professionals are aware that it should be implemented in primary care [60]. However, training needs to be scaled up to ensure that professionals develop the required communication and collaboration skills. As mentioned by Hodorowiwicz et al., [61] organizations are often not able to make direct service staff available for periods of intensive training, and often have limited resources for providing training. As an added challenge, usual training methods often do not result in motivational skills gain. Training and close supervision over longer periods may be needed to support practitioners in attaining motivational interviewing skills and prevent a tendency to revert back to previous routines [62, 63]. There is growing evidence long-term retention of motivational interviewing skills only happens when active and prolonged hands-on experiential learning is used [64]. Live supervision is an approach that can be used directly in clinical settings. In live supervision, the coach provides direct observation and real-time feedback to a trainee conducting an interview and can watch, listen to, and communicate with the trainee via earpiece, computer screen, or by having the trainee step out for consultation. This real-time feedback increases self-awareness and improves clinical skills [65]. 

Another barrier found in our study is the lack of common education tools available to support T2DM self-management. However, multiple high-quality resources and tools are available online from reliable sources, such as Diabète Québec and Diabetes Canada. Learning about these tools should be made a standard component of initial and continuous training of nurses and diabetes educators. There is a need to disseminate this information and provide clear guidelines on how providers should assess their patient level of literacy and use the appropriate tools to optimize self-management education and support. This can take the form of short literacy and learning preferences survey and recommendations to access different websites and/or online learning modules. Furthermore, it is also important to make sure patients are knowledgeable of community resources available to support T2DM self-management. In the province of Quebec, an online and phone free directory of community resources is available for all residents. However, this directory needs to be improved in partnership with patient partners and with regional and local stakeholders to be more comprehensive and facilitate its ease of use. Furthermore, it is also important to improve how patients, in collaboration with professionals, can use effectively this directory to access resources. Finaly, telemedicine tools and improvement of electronic medical records can help the healthcare system address all the above-mentioned QI needs by facilitating patients’ systematic follow-up, interprofessional collaboration and increased empowerment and engagement of patients in the monitoring and improvement of their health and their care. It is important to develop appropriate tools to support primary healthcare teams in the adoption of these technologies in a way that is save and respectful of patients needs and confidentiality.

### Strengths and limitations

This study presents multiple strengths: multiple regions participated to the program and a wide variety of professionals, patients-as-partners and managers were involved in the COMPAS + workshops. Providing feedback using administrative data, engaging participants in reflection on their practice and supporting local QI teams to implement QI strategies contributed to the richness of the data analyzed. Reflexive and problem-solving activities included in the workshops facilitated data collection and the achievement of a certain level of consensus between participants. Ongoing engagement of the project team with partners in each region also improved the team’s understanding of the quality issues experienced. Similar concerns expressed between regions and local services networks increased credibility and transferability of the research results. However, workshops were conducted before the COVID-19 pandemic and some QI priorities may have changed since then, even if the reported results continue to resonate with our project partners involved in reviewing the findings of this study. Patients-as-partners were involved in this QIC; however, using other research methods to engage a greater number of people living with diabetes could also have strengthened this study’s findings. Results of this study does not provide detailed information on the implementation and effects of the QI strategies proposed. Ongoing research on the COMPAS + QIC will allow our team to analyze and publish these results.

## Conclusion

The COMPAS + quality improvement collaboratives led to a better understanding of the challenges related to the delivery of high-quality diabetes care in primary care. Our study also provides insights on how this program helped participants to formulate priorities and strategies at professional and organizational levels to better prevent and manage diabetes. The COMPAS + program shows potential as an important component of an emerging learning health system designed to support continuous learning and improvement in diabetes prevention and management in primary care.

## Data Availability

The datasets used and/or analysed during the current study are available from the corresponding author on reasonable request.

## Abbreviations

BCW: Behavior Change Wheel
BP: Blood Pressure
CDSIC: Chronic Disease Support and Intervention Center
CFIR: Consolidated Framework for Implementation Research
CISSS: Integrated Health and Social Services Centres
DM: Diabetes Mellitus
FMG: Family Medicine Groups
HbA1c: Glycated hemoglobin
INESSS: National Institute of excellence in health and social services
LDL-C: Low-Density Lipoprotein-Cholesterol
LSN: Local Services Networks
MSSS: Quebec Ministry of Health and Social Services
QI: Quality Improvement
QIC: Quality improvement collaborative
T2DM: Type 2 Diabetes Mellitus
USD: United State Dollar
